# Civil Aviation Policies and Practices in Turkey in a Global Context Through Sustainable Aviation

**DOI:** 10.1002/gch2.202300194

**Published:** 2023-11-01

**Authors:** Betul Hande Gursoy Haksevenler, Nazife Umran Temel, Yelda Erden Topal

**Affiliations:** ^1^ Department of Political Science and Public Administration Faculty of Political Science Marmara University Kadikoy Istanbul 34722 Turkey; ^2^ Department of Economics Science Technology Policies Research Center (TEKPOL) Middle East Technical University Ankara 06800 Turkey

**Keywords:** case comparison, civil aviation, document analysis, interview, sustainability, Turkey

## Abstract

The negative environmental effects of global civil aviation growth since the 19th century lead to the emergence of the “sustainable aviation” concept. This study aims to determine the current status of sustainability discussions in developing Turkish civil aviation and its alignment with global sustainable aviation policies. The research data are collected through document analysis and key expert interviews. Document analysis is to review reports on sustainable aviation and to compare two cases of AirFrance‐KLM and Turkish Airlines. Semi‐structured interviews are conducted with key experts from airline companies, public institutions, and subsidiary services in the Turkish aviation sector. The analysis results are presented under the themes of “institutional, conceptual, Turkey's current situation, motivations, role of international organizations, cooperation and coordination, problems and obstacles.” The results show that the Turkish civil aviation industry is enthusiastic about catching up with international standards and is successful in keeping up with the latest technologies. The private sector is leading the sector by targeting global competitiveness. Current deficiencies stem from legislation, incentives, obligations, resources, and environmental awareness. The main contribution is to be the first source and guide for future studies that aim to shed light on policymaking in Turkey as an emerging country case.

## Graphical abstract

There are six sections about sustainable civil aviation in Turkey with a comparison analysis and key expert interviews. After the introduction of sustainable aviation, state‐of‐the‐art, and research design; the results are presented with the current situation, problems, obstacles, and facilitators for sustainable aviation in Turkey, and supported by Air France‐KLM and Turkish Airlines Cases. It concludes with policy proposals for promoting sustainable aviation.

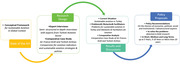

## 1 Introduction

Global environmental problems, which started to be experienced with the industrialization process, have revealed the need for the concept of sustainability. This concept has implications for many sectors and is particularly important for aviation, a rapidly growing industry in the world. Civil Aviation has become quite widespread in terms of volume in the 20th century due to its ability to provide travel opportunities for various purposes, such as the transportation of cargo and passengers over long distances, trade, and tourism. It is predicted that growth will continue in the sector for many more years.^[^ ^1^, ^2^ ^]^ Along with growth, environmental and social costs incurred by industry such as climate change and the impact on the ozone layer, pollution on local and global air quality, noise problems, ground and surface water contamination, and habitat degradation,^[^ ^3^, ^4^ ^]^ have increased significantly. Therefore, sustainability has become as important as safety and security issues in civil aviation in recent years.

According to the Intergovernmental Panel on Climate Change (IPCC) 2022 report, the transportation sector's share in global greenhouse gas (GHG) emissions was 23% in 2019, with the aviation sector accounting for 12% of transportation emissions.^[^ ^5^ ^]^ Despite the COVID‐19 travel restrictions, air traffic trends were still moving upward, increasing at an annual rate of 3.3% between 2010 and 2019. However, emissions from international air travel fell by 45% in 2020 compared to 2019 due to the pandemic. Addressing the necessity of reducing carbon emissions on a global scale, the aviation industry has made significant investments in engine technology. Nevertheless, major changes in aircraft configurations are not expected until at least 2037, making the decarbonization of the aviation industry a challenging task.^[^ ^5^ ^]^ Policymakers are now urged to focus on sustainable growth, prompting international organizations and air transportation sector stakeholders to scrutinize GHG emissions.^[^ ^6^, ^7^ ^]^

Differences between policy and practice regarding sustainability^[^ ^8^ ^]^ arise from the difficulty of conducting a deep analysis to strike a real balance between economic, social, and environmental factors. In current practices, sustainability is often addressed through sectoral developments, and the aviation sector stands out as one of the most challenging to implement sustainability.^[^ ^9^, ^10^, ^11^ ^]^ A mutually accepted and agreed definition of sustainability in the aviation industry is still lacking, and the concept only covers a narrow perspective emphasizing eco‐efficiency.^[^ ^12^ ^]^ Therefore, it is crucial to examine the obstacles to sustainability in the aviation sector and investigate how they can be overcome. Success in this difficult policy area could provide valuable lessons to promote sustainability in other sectors.

Based on this context, the aim of this study is to examine the level of sustainability adoption in civil aviation in Turkey and identify ways to improve it. To understand the current state of sustainability in Turkish civil aviation, we analyzed key documents and reports and complemented our analysis with expert interviews. The study's findings contribute to revealing the necessary policy actions for the development and enhancement of sustainability in Turkey's civil aviation sector, addressing the sustainability discourse not only at the local level but also on a global scale.

## 2 Literature Review

Given the global and complex nature of environmental problems, addressing them requires comprehensive and integrated approaches involving national and international cooperation, as well as public, private, and civil initiatives.

Studies on sustainable aviation primarily focus on GHG emissions,^[^ ^13^, ^14^, ^15^ ^]^ climate change effects of the aviation industry,^[^ ^16^, ^17^ ^]^ and fuel savings (through developments like new generation aircraft technology).^[^ ^18^, ^19^ ^]^ These studies mainly underlined the multidimensional nature of the “sustainability” concept in the aviation sector. Chao et al.^[^ ^13^ ^]^ elaborated US Airlines as a case study for sustainable aviation by mainly focusing on the potential impacts of policy tools by the International Civil Aviation Organization, Carbon Offsetting and Reduction Scheme for International Aviation (CORSIA), on USA greenhouse gas emissions. The results show that with the technological improvements (of using a combined model of airlines operations) and environmental friendly fuel (Sustainable Aviation Fuels‐SAF), the U.S. aviation sector is projected to reduce GHG emissions by 2050. Here, the importance of two economic factors are highlighted to reach this target: The price of petroleum‐based aviation fuels and the growth rate of the carbon price. In another study on the role of a specific policy tool, sustainable aviation fuels, for decarbonization of the aviation sector, Malina, Abate, and Schlumberger^[^ ^14^ ^]^ pointed out that the aviation industry and air transport are indispensable pieces in the global economic welfare puzzle, and to realize a decarbonized future by increasing the role of SAF usage, the report proposes a multidimensional solution package that includes economic aspects (of demand change for airport transfer), technological aspects (of improvements in the aircraft systems), managerial and administrative aspects (of improvements in flight and ground operations), and use or environmental friendly solutions (of SAF consumption in air transport; ref. [14], p. xviii). The main message given by the paper is the strong communication and collaboration between policymakers, industry leaders, and financiers to address the economic and technological problems in the sustainable aviation industry. Another study underlying the multifaceted nature of sustainable aviation is Ryley, Baumeister, and Coulter^[^ ^15^ ^]^ covering a systematic literature review on the causes of climate change emerging from the aviation sector. The main outcome of the paper is the need for additional progress in policy making particularly in the realms of aviation adaptation planning and the social justice among transportation users. In their systematic literature review on noise topic in airports, Rodríguez‐Díaz, Adenso‐Díaz, and González‐Torre^[^ ^16^ ^]^ elucidated the main discussions on the effects of aviation on climate change and the solutions persuaded by the aviation industry to cope with climate change. The main conclusion derived by the analysis is that rather than the chemical, engineering, or other physical factors, perspectives and actions of aviation stakeholders and their response to climate change policies drive and limit specific adaptation pathways of the aviation industry to climate change. On aviation noise impact, Leylekian, Covrig, and Maximova^[^ ^17^ ^],^ in their edited book as an output of the ANIMA project (Aviation Noise Impact Management through Novel Approaches), elaborated on the impacts of aviation noise on human beings, annoyance, quality of life, and health in addition to well‐ known issues such as acoustic factors creating and affecting noise pollution emerging from aviation operations. The topics tackled in the book are proof of the multidimensional appearance of sustainability in the aviation sector. Both Shahriar and Khanal^[^ ^18^ ^]^ and Cabrera and de Sousa^[^ ^19^ ^]^ investigated how sustainable aviation fuels (SAFs) can be used, disseminated and supported in the aviation industry in addition to motivations and obstacles to use; both papers concluded that the use of alternative fuels (to fossil fuels) becomes a prerequisite to decrease the carbon footprint of the air transportation and this diffusion must be supported by economical aspects (i.e., government incentives), technical aspects (of new technology development to increase the efficiency of the fuel), and political aspects (alignment of national and international regulatory frameworks of aviation). However, these developments and suggestions alone cannot effectively address the sustainability challenge.^[^ ^20^, ^21^, ^22^, ^23^ ^]^ Gössling and Lyle^[^ ^20^ ^]^ claim that it is possible to decrease aviation emissions, but achieving this goal necessitates policy measures either on a national scale or within regional entities like the European Union. Singh, Rana, Hamid, and Gupta^[^ ^21^ ^]^ extensively analyzed different decision‐making levels to gain insights into the possibilities and prerequisites associated with promoting sustainability in aviation by tactical level and operational level decisions. The study concluded that decisions at the tactical level had the highest impact on reducing aviation fuel consumption, followed by decisions at the operational level and decisions at the strategic level. According to the results, the mid‐level stakeholders such as aircraft manufacturers and airline companies play a critical role in decision‐making, therefore, the role of these stakeholders is suggested to be strengthened in order to connect the lower and upper levels and activate them. In their article, Leal Filho, Ng, Sharif, Janová, Özuyar, Hemani, Heyes., Njau, Rampasso^[^ ^24^ ^]^ investigated the effects of the global aviation sector on climate change within the framework of global tourism. In this context, they conducted a bibliometric analysis of the literature by scanning 772 articles and additionally examined 20 commercial airline companies that pursue sustainability policies in line with the Paris Agreement goals. The results show that companies made some self‐regulation to reduce carbon emissions, but they were not sufficient in terms of voluntary compliance and commitment to long‐term goals. As a solution, they proposed strategies that include creating compatible sustainable infrastructures among the airport networks in international tourist destinations and ensuring adaptation to sustainability targets through a radical transformation.

Each aspect of the multidimensional “sustainability” concept should progress in balance with each other. The idea of environmental sustainability becomes prominent as international aviation authorities consider economic growth unsustainable unless it can be attained.^[^ ^25^ ^]^ The concept of sustainable aviation is introduced by the necessity of taking precautions against this threat and these efforts are to achieve a cleaner, quieter, and smarter aviation industry in the future. It represents a long‐term strategy. This strategy is relevant to the fight against climate change, noise pollution, and weather pollution by international aviation authorities for global well‐being.

While many international organizations prioritize policies on safety and security in civil aviation, only a few focus solely on environmental sustainability. The main policy‐making body in this regard is the International Civil Aviation Organization (ICAO), a civil aviation organ of the United Nations (UN). ICAO has emphasized the three pillars of sustainability: i) state action plans, ii) sustainable alternative fuels for aviation, and iii) market‐based measures, which consider the social, economic, and environmental aspects of sustainability.^[^ ^26^, ^27^ ^]^ ICAO develops standards, recommended practices, and policies concerning noise pollution, emissions and air quality, operational improvements, aircraft and fuel technologies, and cost‐effective markets. One of its primary goals is to reduce GHG emissions from civil aviation by 50% by 2050, compared to 2005 emissions.^[^ ^26^ ^]^ Additionally, the Paris Agreement (2015) of the United Nations aims to reduce carbon emissions from aviation by 50% between 2020 and 2050 compared to 2005 levels. At the regional level, the European Commission's Emission Trading System has included taxation for each unit of emission originating from aviation since 2012. Moreover, the Green Deal,^[^ ^28^ ^]^ implemented in the European Union (EU) since 2020, aims to make Europe the world's first climate‐neutral continent by 2050, with a specific goal of reducing carbon emissions by 55% by 2030 compared to 1990 levels.^[^ ^29^ ^]^ The “Single European Sky” application also enforces taxation for carbon emissions within EU member countries and flights to these countries.

In the above‐mentioned global context, the aviation sector in Turkey, the subject of this study, has experienced significant development, driven by its favorable geographical location and increasing passenger and flight traffic. Despite the pandemic, the number of passengers surged by 57.4% in 2021, reaching 128.5 million after the normalization process, compared to ≈82 million in 2020.^[^ ^30^ ^]^ According to EUROCONTROL (The European Organisation for the Safety of Air Navigation)'s traffic statistics, Turkey ranked fifth in Europe in 2021. Additionally, Turkish Airlines, the country's largest airline, secured the second position among the airlines with the most flights in 2021.^[^ ^31^ ^]^ Conversely, according to the Inventory Report published by the Turkish Ministry of Environment and Urbanization in 2021, 93% of emissions originating from transportation in Turkey come from the highway, 4.5% from the airway, 1.1% from the seaway, 0.5% from the railway, and 0.9% from other alternative modes of transport.^[^ ^32^ ^]^

Given these data, it becomes crucial to assess the extent to which sustainability policies are embraced in this rapidly growing sector. Notably, there is a limited number of studies on the sustainability of the aviation sector at a global level as well as in Turkey.^[^ ^33^, ^34^, ^35^, ^36^ ^]^ It is seen that the solution suggestions for ensuring sustainability in aviation are generally environmentally based and focus on carbon emission reduction in Turkey, as in other examples around the world. For emission reduction, fuel and aircraft technologies and the sustainability of airports were emphasized. However, as highlighted in this research, there is a noticeable scarcity of studies that advocate for a comprehensive approach to the issue and highlight the shortcomings attributable to policymakers.

In their study, Bakır, Bal, and Akan^[^ ^37^ ^]^ systematically examined the rapidly developing Turkish civil aviation sector, its strengths, weaknesses, opportunities, and threats (SWOT) by making a SWOT analysis. Upon concluding the study, which assessed the present state of the industry, it was determined that the paramount factor influencing the sector is its tourism potential. In their study, Selimoğlu and Çalışkan^[^ ^35^ ^]^ argued that the economic, social, and environmental dimensions of sustainability in aviation should be addressed in a more integrated manner, and pointed out the lack of sustainability reporting in airlines. At the end of their study on the status of sustainability reports in the Turkish aviation industry, it was determined that only one airline out of 13 airlines operating in the Turkish civil aviation sector had a sustainability report, and in addition, no airport management company or ground handling company published sustainability reports in the sector. Karakuş. Polat, and Karşıgil^[^ ^36^ ^]^ in their study to understand the perception of environmental sustainability in the Turkish aviation industry; concluded that, in general, all stakeholders in the sector have the knowledge to a certain degree about environmental sustainability, but the lack of awareness in society and the inadequacy in legal regulations negatively affect the diffusion. He envisaged the need for airline companies, NGOs, and all other stakeholders to develop common ideas and work to increase sustainable practices as a solution, and attributed the development of the sector in this sense to providing community support.

This paper contends that tackling the sustainability issue solely from an environmental dimension is insufficient, and a more comprehensive approach is needed, involving a network chain of civil and non‐sector actors, policymakers, institutions, and organizations in the sector, all working together to achieve the common goal, as suggested by Payán‐Sánchez, Plaza‐Úbeda, Pérez‐Valls, and Carmona‐Moreno.^[^ ^38^ ^]^

## 3 Research Design

The research data for this study were collected from both primary and secondary sources. The research design utilized desk research for document analysis and a qualitative research method involving semi‐structured interviews. Document analysis was done to compare methodologies and results of two sustainable aviation practices at a global level by the institutional reports and strategy documents of prominent examples of Turkish Airlines and AirFrance‐KLM. Subsequently, semi‐structured in‐depth interviews were conducted with key experts in the Turkish aviation sector as the groups of airline companies, public institutions, and subsidiary services. Initially, comprehensive desk research was conducted to review reports from international and national institutions on sustainable aviation and gather relevant figures from statistical databases. This allowed us to identify the application areas of sustainability in the sector, including the targets set by civil aviation authorities and compliance obligations. Subsequently, semi‐structured in‐depth interviews were carried out with key experts from prominent institutions and companies in the Turkish aviation sector. The transcriptions of these interviews and the elaboration of institutional documents, such as strategy papers and company plans, served as supplementary data for analysis. The use of interview methods with key stakeholders is a widely adopted practice in aviation literature,^[^ ^39^, ^40^ ^]^ offering insights into stakeholders' perspectives on the adoption of sustainability in civil aviation, which is a relatively understudied field.

The sampling frame for this study includes actors and stakeholders in the Turkish Civil Aviation Sector. Due to its structure, the civil aviation sector involves a diverse range of national, international, sectoral, and sub‐sectoral actors collaborating and conducting business together. In Turkey, the main actor groups are as follows: i) Airline Companies—Operating Actors, ii) Public Institutions—Regulating Actors, iii) Aviation Service Businesses—Subsidiary Services, and iv) Non‐Governmental Organizations (such as Associations and Trade Unions)—Supporting Actors. The sectoral actors (which are directly working in the civil aviation sector) are airline companies and regulating public institutions. The sub‐sectoral actors (which are working on sustainable aviation but not operating directly within the sector) are subsidiary service actors (such as consultancy companies on sustainable aviation) and non‐governmental organizations (such as ICAO: Global Coalition for Sustainable Aviation and ICSA: International Coalition for Sustainable Aviation, IATA: The International Air Transport Association, EUROCONTROL: The European Organization for the Safety of Air Navigation)

To support the documentary research and observe network interactions in this multi‐actor sector, the research design included key participants from the groups of airline companies (referred to as “sectoral”), public institutions (referred to as “public”), and subsidiary services (referred to as “sub‐sectoral”) (**Table** **1**).

**Table 1:** Profile information of the interviewed participants.

Participant	Actor	Type Institution	Position	Experience (Years)
P1	Sectoral	Full Service Provider Airline (High Cost Airline)	Mid‐Level Manager	11
P2	Sectoral	Low Cost Airline	Senior Manager	9
P3	Sectoral	Airport Management	Mid‐Level Manager	12
P4	Sub‐sectoral	Sustainability Consulting Company	Managing partner	14
P5	Public	Public Institution	Aviation Specialist	7

To select interviewees, we used purposeful sampling^[^ ^41^ ^]^ as the sampling technique. This sampling approach focuses on engaging with actors who are expected to possess the most extensive insights and allows us to obtain critical information from the most knowledgeable informants about the sector. While random sampling is rooted in statistical probability theory, providing researchers with the opportunity to draw statistically valid and confident empirical generalizations about a population through the selection of a random and representative sample, in purposeful sampling the researcher‘s intentional choice of the data source gives more accurate and to‐the‐point information about the research focus in the qualitative data collection.^[^ ^42^ ^]^ Therefore, in our research context of sustainable aviation in Turkey, as the information‐rich informants we directly communicated with prominent actors in aviation as the representatives from the aviation stakeholders grouped under high‐cost airline, low‐cost airline, and airport management in addition to a public institution responsible for critical regulatory operations in the sector, and a private sector company providing sustainability services to airlines. This selection provided a comprehensive picture of the adoption dynamics of sustainable aviation in the sector (Table 1).

The interview form included ten questions covering topics such as “participant profile,” “conceptual approach to sustainability,” “corporate approach to sustainability,” “Turkey's sustainability performance in the sector,” “the main motivational source for sustainable aviation policies and practices,” “the role of international organizations (such as the United Nations, European Union),” “public‐sector cooperation and coordination,” and “problems and deficiencies in the industry.” The interviews were conducted between October 14 and December 23, 2021, using both online and face‐to‐face formats. The profile information of the participants was coded and presented in Table 1.

For the analysis of the data, we used the “analytical framework approach” as described and used in a former study that included comprehensive qualitative data of interviews on sustainability.^[^ ^43^ ^]^ There are three primary methods available for describing and structuring raw data, which are essential for organizing and presenting it effectively. These methods are known as the “Storytelling Approach,” the “Case Study Approach,” and the “Analytical Framework Approach,” as described by Patton (ref. [42], p. 439). In the Storytelling Approach, the primary objective is to provide a chronological and comprehensive account of the narrative, starting from its inception and progressing through its entire development. The Case Study Approach, on the other hand, involves analyzing individual cases within the data, which can include individuals, significant events, or the specific settings where data is generated, such as places or locations. In the Analytical Framework Approach, the focus is on describing data by examining the underlying processes, themes, patterns, key issues, or sensitizing concepts that hold significance within the research area. This approach often involves organizing responses to interview questions, particularly when a standardized interview format is utilized.^[^ ^42^ ^]^

## 4 Results and Discussion

For the analysis of sustainable aviation in Turkey, the research approach involved two main components: document analysis and key expert interviews.

In the document analysis, the focus was on understanding the current state of sustainable aviation in Turkey. The researchers examined various documents, reports, and publications from international and national institutions to gain insights into sustainable aviation practices, identify existing problems, and recognize obstacles and facilitators for sustainable solutions. This comprehensive desk research aimed to provide a solid foundation for further analysis.

The second component of the research involved key expert interviews. Through semi‐structured interviews with experts from prominent institutions and companies in the Turkish aviation sector, the researchers delved deeper into the sustainability issues identified in the document analysis. These in‐depth interviews allowed the researchers to gather valuable perspectives from key stakeholders and understand the challenges and potential solutions from their viewpoints.

By combining the findings from the document analysis and key expert interviews, the researchers aimed to identify critical sustainability problems in the aviation sector. This holistic approach enabled them to propose policy implications based on the research outcomes and determine the main potential solutions for addressing the sustainability challenges. Moreover, the limitations identified during the analysis could help provide a more comprehensive understanding of the complexities involved in achieving sustainable aviation in Turkey.

Overall, this research aimed to offer a thorough examination of sustainable aviation in Turkey, combining insights from both primary and secondary sources. By analyzing documents and conducting key expert interviews, the researchers sought to identify practical solutions and develop policy implications that could contribute to the advancement of sustainable practices in the aviation sector.

### 4.1 Sustainable Aviation in Turkey: The Current Situation

Turkey's current situation in sustainable aviation is examined under four key headings: institutionalization, conceptualization, source of motivation, and sector‐state cooperation.

In terms of institutionalization, the research reveals that having a dedicated sustainability unit within institutions is a crucial step for executing sustainability studies. However, it was observed that the level of institutionalization in Turkey regarding sustainability was not sufficient, and steps towards institutionalization were only recently taken based on the interview data.

The first initiative for institutionalization in the Turkish civil aviation sector occurred in 2019 when Turkish Airlines established a specific department to manage, implement, and monitor its sustainability processes and operations. In comparison, Air France‐KLM, considered a successful example in world civil aviation, started preparing sustainability reports back in 1996/1997. Although Turkey started this process relatively late, significant progress has been made in recent years, especially in the follow‐up and implementation of new technologies, as reported by key experts.

Conceptually, Turkey's approach to sustainable aviation considers its national and international priorities and aims to maintain political balances in response to developments. A study by Gelirli and Yasar^[^ ^33^ ^]^ investigating sustainability practices in Turkey's aviation sector found that the economy is given higher priority compared to the environmental and social dimensions of sustainability. However, the interviewees emphasized that true success can only be achieved if all dimensions of sustainability are balanced and addressed equally.

The main motivation for sustainable aviation studies in Turkey arises from competition in the international structure of the sector and the expectations of investors and stakeholders. Companies also act with social responsibility due to these expectations. Additionally, international obligations, agreements, and sanctions play a significant role as a source of motivation. Organizations such as the EU and the UN are particularly influential in creating motivation and pressure. The EU imposes mandatory sanctions on airlines operating in its airspace to contribute to environmental sustainability and protect its market competitiveness. As Turkey has trade relations with the EU, it is obligated to comply with policies like the “Border Carbon Regulation.” Furthermore, the UN plays a significant role in setting international standards in civil aviation, influencing decisions and practices within the sector.

In summary, Turkey has made progress in recent years in terms of sustainable aviation, but there is room for further institutionalization and balance among economic, social, and environmental dimensions. The motivation for sustainable aviation is driven by international competition, investor and stakeholder expectations, and compliance with international obligations and agreements. The EU and the UN play essential roles in shaping sustainability practices within the Turkish aviation sector.

Another significant aspect of the discussion is the dimensions of sector‐state cooperation and the question of who should take the lead. The civil aviation sector regularly collaborates with public actors on matters such as determining flight routes and establishing regulations. However, when it comes to complying with global standards, it is observed that the private sector often takes the lead. The sector proactively adopts some practices (such as Research and Development (R&D) and investments for SAF production and efficient fuel consumption, new technological developments for operational efficiency) that are not mandated or directly regulated by public authorities but are required by international civil aviation bodies or driven by competitiveness.

During the interview analysis, different opinions were observed among the participants regarding who should be the pioneer in sustainable aviation. One sector representative emphasized the importance of state leadership, while the public representative argued that sectoral leadership should drive developments, with the state supporting and facilitating the process of enacting necessary practices.

The analyses are presented under the themes derived from the research and labelled classifications based on qualitative data of the interviewees. For the data analysis, as described in “3. Research Design” part, we used the “Analytical Framework Approach” in arriving at those themes. Under the context of this approach, we used pattern recognition by benefitting from inductive “content analysis,” which refers to the “qualitative data reduction and sense‐making process of distilling a substantial amount of qualitative information to uncover fundamental consistencies and to derive meaningful insights” (ref. [42], p. 453). In this content analysis, we followed the technique of “open coding” “to break the data apart and delineate concepts to stand for blocks of raw data” (ref. [44], p. 198). In the process, these blocks of raw data emerged as the main themes of “Institutional approach to sustainability,” “Conceptual approach to sustainability,” “corporate approach to sustainability,” “Turkey's sustainability performance in the sector,” “the main motivational source for sustainable aviation policies and practices,” “the role of international organizations,” “public‐sector cooperation and coordination,” and “problems and deficiencies in the industry.” **Table** **2** provides a summary of the current state of civil aviation in Turkey in line with these classifications and themes.

**Table 2:** Current situation in sustainable aviation in Turkey.

Theme	Characteristics	Finding (F) from the Document Research & Interviews Supplementary Comments and Quotations from interviewees (Participant Code in Parenthesis)
Institutional approach to sustainability	Present Not present	**F1: Long history and background of institutional sustainability studies** “Although a relevant department on sustainability has existed since 2020, sustainability studies have been carried out for many years. A sustainability report has been prepared in line with investor expectations in order to be included in the sustainability index created in Borsa Istanbul since 2014” (P1). **F2: Very recent conceptualization and labelling of “institutional sustainability”** “Sustainability studies are handled as a sub/sub‐field within the current institutional structure. The institutional approach to the subject is just beginning to take its place in the general institutional structure. Rapid steps have been taken on the subject, especially in the last 2–3 years” (P2).
	**MAIN CONCLUSION**: Although sustainability studies in the aviation sector have been conducted for a long time, they were previously considered as a subdomain or a subset of the broader institutional studies. In other words, sustainability was not treated as a separate and unique field but rather as a specialized area within the existing institutional framework. However, there has been a recent shift towards giving sustainability a more prominent and independent position within the overall institutional structure. This indicates a growing recognition of the significance of sustainability in the aviation sector and the need to address sustainability challenges with more focused and dedicated efforts. While there has been a long history of sustainability efforts, there has been a recent push towards a more formal and institutionalized approach to sustainability. This shift reflects the sector's growing commitment to addressing sustainability challenges and adopting sustainable practices.	
Conceptual approach to sustainability	Environmentally, economically, socially balanced Environmentally oriented	**F3: Balanced approach rather than picking one of the sustainability dimensions as the most important and critical** “No dimension can surpass the other; when these three dimensions are not balanced, true sustainability cannot be achieved. Prioritizing the economic dimension over the environmental dimension may lead to unsustainable financial practices in the long run. Similarly, solely focusing on the environmental dimension without considering economic viability could hinder the long‐term sustainability of initiatives. The key to sustainability lies in finding harmony among all dimensions, recognizing their interconnectedness and interdependence. Striking the right balance ensures that economic, social, and environmental aspects support each other, leading to genuine sustainability in the aviation sector.” (P1). **F4: Sustainability means mostly environmentally friendly and low carbon intensive solutions and approaches** “It seems that in Turkey, there is a focus on the environmental aspect of sustainability. This is relatively easier to address, with initiatives aimed at reducing fuel consumption, waste generation, and overall resource consumption in the environmental field. However, it proves to be more challenging to address the social side of sustainability, particularly in terms of the well‐being of employees, suppliers, customers, and business partners. Achieving sustainability requires improvement in every aspect, and neglecting one side will leave the other lacking” (P2). “Although this situation is related to the companies' point of view, especially in recent years, there has been a growing realization of the importance of the carbon issue. As a result, sustainability is now being considered with a stronger environmental focus” (P3).
	**MAIN CONCLUSION**: Environmentally‐oriented sustainability is more dominant, however, the overall approach should be balanced	
Turkey's sustainability performance in the sector	Successful Open for improvement	**F5: Successful in following, implementing, synthesizing and searching for new technological developments** “Turkey is quite successful, especially in terms of adopting new technologies. It excels not only in quickly adopting and utilizing these technologies but also in developing innovative approaches, such as biofuel management and synthesizing existing technologies to achieve reductions in various areas.” (P4). “Their investments in state‐of‐the‐art aircraft, which provide less noise and emissions consumption, and the relatively young average age of their fleets are contributing to their success in this context.” (P1, P2). **F6: Turkey is open to improvements and new developments in sustainability approaches, but it does so at a slow pace, which creates the main point of difference from other examples**. “According to my estimation regarding the environmental aspect in aviation, it will take at least 6–7 years for us to reach the desired level. We need to prioritize the work we do until we achieve these goals. While the EU completed its growth in aviation in the 1990s, the process is still ongoing at a significant level in Turkey, resulting in a different approach to sustainability” (P5)
	**MAIN CONCLUSION**: Turkey's performance is successful and open to improvement in all aspects of sustainable aviation.	
Main motivational source	Competition World Standard Service Obligation‐Volunteer	**F7: Sensitive and aware customers force sustainable aviation practices**. “Although sensitive customer and investor expectations result in additional costs during this process, they compel companies to prioritize sustainability. It is imperative for the aviation industry that the customer is highly conscious and gives priority to this issue, particularly with the emergence of the concept of ‘flying shame” (P1).
		**F8: Compatibility with the world sustainable aviation standards is a MUST** “Due to the international nature of the sector, the source of motivation adheres to world standards” (P3). “The necessity of adapting to international regulations such as CORSIA is the main motivation source” (P1). “Being subject to certain international obligations, especially the Paris Agreement, has now become an inevitable competitive element for the sector. For Turkey, it has evolved into a matter of prestige, driven by political reasons” (P5). **F9: Volunteered as much as forced by environmental regulations about carbon emissions**. “The country should implement regulations internally and integrate them into the economic system, recognizing that carbon bears both environmental and social costs. In other words, carbon carries a price and serves as a risk factor in all microeconomic decisions, such as expenditure, determining production systems, production levels, and regulatory considerations. Additionally, it compels the adoption of a “low carbon economy.” On the other hand, well‐known brands that proactively embrace this process, even if they are not bound by emission or carbon regulations, demonstrate a voluntary commitment” (P4).
	**MAIN CONCLUSION**: The sources are sensitive and aware customers, compatible with world sustainable aviation standards, and motivated by both voluntary commitment and compliance with environmental regulations	
Role of international organizations (EU‐UN)	Compliance with EU Regulations UN Standards EU Sanctions (CBAM) Paris Agreement	**F10: EU Emission Trading System (ETS) Calculations, requisites, competition rules and funding opportunities** “As the decisions taken in the EU fall within the scope of flight operations, they are binding and require carbon calculations as part of the EU ETS. Consequently, they receive carbon credits” (P1). “While the EU's primary goal is to maintain the competitiveness of its own market, it remains a political community that takes the lead on the issue and establishes connections with the international sector. According to the UN, this is evident in the targets it has set and the funds it provides.” (P2). **F11: Global and local compatibility via UN Standards** “ICAO, affiliated with the UN, establishes the framework for global aviation management, international standards, and enables their implementation worldwide. In terms of CORSIA, carbon offset studies are of significant importance to align with global standards” (P2). **F12: Carbon Border Adjustment Mechanism** With the implementation of the Carbon Border Adjustment Mechanism (CBAM) process, the EU will introduce carbon pricing for some goods coming from non‐EU countries that trade with it, this practice will force Turkey, so the EU's coercion will be felt more quickly in the industry and business world (P3, P4). **F13: Included as a partner in Paris Agreement** “Although the Paris Agreement is not compelling, it must be involved in order to maintain international balances” (P4).
	**MAIN CONCLUSION**: Compatibility to International and EU Level Obligations adds value to local sustainability applications	
Public‐sector cooperation and coordination	Partnership Who is/Should be the Pioneer Public‐consumer effect	**F14: Cooperation between public and private sectors is promoted and supported** Industry and government cooperate. The sector provides a conducive environment for establishing close interactions with the public. (P1, P2, P4, P5). **F15: Sector‐led developments and implications for sustainability** “The industry holds a prominent position owing to its global nature. Nevertheless, the state and the sector are closely intertwined in the audit and support procedures” (P4). “Given that the resources in the sector surpass those available in the public sector, making the sector a pioneer is imperative.” (P5). “The public should play a leading role and formulate a strategy, similar to the coercive role of international organizations. By adopting a top‐down approach, companies in the sector will be encouraged to contribute with a global vision, thereby yielding beneficial outcomes.” (P2). **F16: The main source of pressure for sustainable practices comes from consumers who exert influence on suppliers**. “The public and consumers should encourage themselves; the most important aspect is consumer pressure. As voters, the public should influence politicians, the state, and the governing political power. Companies should also contribute by making sustainable brand choices and supporting non‐governmental organizations.” (P4).
	**MAIN CONCLUSION**: Sector‐led and cooperative sustainable solutions are forced by environmentally sensitive consumers	

In this context, the analysis presents a comprehensive view of sustainable aviation in Turkey, taking into account various perspectives from both the public and private sectors. The debate on sector‐state cooperation and the pioneer's role is a crucial aspect to consider in the journey towards achieving sustainable aviation in the country. By examining these themes and classifications, the research aims to shed light on the challenges and opportunities for sustainable aviation in Turkey and identify potential areas for improvement and policy implications.

### 4.2 Problems for Sustainable Aviation in Turkey and Obstacles and Facilitators for Solution

When examining the problems related to ensuring sustainability in civil aviation, the obstacles to their solutions, and the supporting factors, the priority issue is identified as “the lack of awareness.” As environmental awareness becomes ingrained in society's culture, there will be increasing pressure on companies to adopt environmentally friendly practices. It has been observed that aviation operations will become more environmentally friendly as politicians respond to voters' demands and brands align with the expectations of consumers and investors. However, in Turkey, environmental awareness seems to be top‐down rather than bottom‐up, as the sector's international structure propagates environmental culture to society.

During the interviews, an important reason cited for the deficiencies in the process was financial constraints. The study's findings confirm that sustainability practices are more costly for growing sectors and developing countries. Literature studies indicate that the cost of such practices is lower in mature markets like the EU, where earlier steps were taken.^[^ ^25^ ^]^

Moreover, interviews revealed that the processes of establishing, enacting, and implementing policies regarding sustainability in civil aviation are progressing slowly. Participants emphasized legal obligations and the inadequacy of incentives, highlighting the significance of public‐sector cooperation. Some deficiencies in state incentives were identified, leading to the need for monitoring developments and implementing legal obligations to standardize practices and balance the increasing costs of developing technologies in the sector. The perception and understanding of sustainability will only become effective with increased cooperation between stakeholders. While companies in the Turkish aviation sector take steps toward social and environmental responsibility and competitiveness, the impact of these steps will remain limited without support from legal processes. Serious deficiencies in national legislation on greenhouse gas emissions were observed in Turkey compared to EU countries. Nationally regulated entities are encouraged, albeit to a limited extent, to identify, report, and reduce emissions, but they are not subject to sanctions. Though monthly emission values of airline companies have been reported since 2015, there are no current plans to switch to the emission trading system due to the associated cost.

Upon examination of opinions, participants perceived Turkey and Turkish civil aviation as successful in adapting to the process despite the need for further policy development and improvements. In Turkey's rapidly growing aviation sector, developments related to sustainability are closely monitored, and efforts are made to foster environmental awareness, which is disseminated from the sector to society.

The problems identified for sustainable aviation in Turkey, along with the obstacles and facilitators, are presented in **Table** **3**.

**Table 3:** Problems for sustainable aviation in Turkey and obstacles & facilitators for solving the problem.

Theme	Problem	Obstacles	Facilitators	Supporting Quotations from interviewees
Economic	Difficulties in implementation of EU ETS Systems	More costly sustainability practices for growing industries and developing countries than established sectors and developed countries.	* Keeping regular and detailed periodic emission reports of airlines *Performance evaluation based on periodic reports	“We are good in terms of technology, but we probably have a problem with financing, so banks have just started to get involved in these businesses. Therefore, to enhance the flow of financing and encourage more investors, it is necessary to establish new financing mechanisms. These mechanisms can facilitate the transition of funding to such businesses and make it easier for investors to participate.” (P4) “The EU completed its aviation growth in the 1990s, but aviation in Turkey continues to experience significant growth. As a result, we are currently not considering implementing this system” (P5)
Political	Compatibility to international Agreements (such as Paris Agreement, Green Deal)	Lack of legal obligation at the national level	Preparation of legal regulations at the national level	“There are not enough legal requirements. When something is legally mandated, you have to comply with it, and you act accordingly. However, in many cases, we strive to implement practices even when we have no legal obligation to do so (P1)
Social (at the aggregate or individual level)	Lack of awareness and mindfulness about sustainable aviation practices	Deficiencies in increasing the amount and pressure of legal obligations and supports/incentives	*Presence of necessary motivation and awareness in leading organizations *Integrated approach to sustainability *To be open to improvement in terms of increasing national perspective and motivation in Turkey.	“The area where we lag behind is primarily in terms of responsibility, awareness, and control. Unfortunately, self‐regulation concerning the environment does not work very effectively in Turkey.” (P4)
Infrastructural/Institutional	Lack of human resources	*Lack of specifically trained experts *Treating the sustainable aviation implications as a by‐product and sub‐domain of main aviation tasks and implications	Leading organizations and airlines are eager to adapt human resource strategies for sustainable aviation	“The unit requires at least ten more people to make a significant impact, allowing everyone to specialize in their respective areas. However, under the current circumstances, it is not feasible. Therefore, we need to reevaluate and prioritize certain tasks to make the best use of available resources." (P5)
	Cooperation between the public and private sector	need to be development, still in the beginning phase.	Motivation needed for specific solutions, supports and subsidy schemes. Suitability of the aviation industry to maintain one‐to‐one and regular relations with public institutions.	“We need to prioritize tasks accordingly. Therefore, if there is something urgent, it is better for the industry to guide us in that direction.” (P5) “For example, sustainable aviation fuel: Is there an investment plan regarding this in Turkey, and will there be incentives for the sector? These issues should be brought to the agenda, discussed openly, and kept on the agenda for further consideration” (P2)

### 4.3 Case Studies of Air France‐KLM and Turkish Airlines

In order to compare the methodologies and the results to make sure that the outcomes are comparable and up to the international standards, the cases of Air France‐KLM and Turkish Airlines are compared.

#### 4.3.1 Air France‐KLM

In 2014, Air France (headquartered at Paris Charles de Gaulle Airport) merged with KLM Royal Dutch Airlines (headquartered at Amsterdam Schiphol Airport) while preserving their individual identities and commercial brands and became Europe's largest airline group. The group's main goal is to become an aviation champion in Europe while taking a pioneering role in sustainable aviation. The Air France‐KLM group has been a leader in the field of sustainability within the aviation industry for many years. Since 1999, the group has been listed on the Dow Jones Sustainability Index (DJSI), which assesses publicly traded international companies separately in governance, environmental, and social sustainability areas. The group was first listed on the DJSI in September 2005 and maintained its leadership position until 2016. Currently, it continues to rank at the top of this index, which has become a reference point for investors incorporating sustainability into their portfolios and announces industry leaders in sustainability every year, maintaining its leadership position among its European counterparts.^[^ ^45^, ^46^ ^]^

Air France‐KLM Group has been a member of the United Nations Global Compact since 2003, contributing significantly to the Sustainable Development Goals, and is committed to being one of the leaders in a more sustainable aviation industry. As a result, since November 2022, it has been one of the first European airline groups with a decarbonization journey approved by the Science Based Targets initiative (SBTi), a collaboration between the United Nations Global Compact, the World Resources Institute, and the Worldwide Fund for Nature.

Every 2 years, the Group conducts prioritization analyses to reassess key areas that are central to its operations and integrates the results into its strategy. According to the prioritization analysis conducted in 2021, Customer Satisfaction, Fleet Development, CO_2_ Emissions Reduction, Financial Performance, and Safety (flight, health, and security) are ranked as the top priority areas. Within this framework, stakeholders continuously monitor strategic priorities that can impact the long‐term growth of the industry, including the acceptability of aviation's environmental impacts, the use of sustainable alternative fuels, changes in customer behavior, control of the carbon reduction path, and fair competition among airlines.

Air France‐KLM Group has set a decarbonization goal within its “Sustainability Objective” program, which is based on three main pillars aimed at reducing environmental impacts. The first pillar involves renewing and modernizing its fleet with the latest generation aircraft, resulting in 20–25% less CO_2_ emissions compared to previous aircraft models. The second pillar is the use of alternative fuels, which can play a significant role in decarbonizing aviation, with potential reductions of up to 80%. The third pillar focuses on increasing operational efficiency, which includes implementing procedures that limit fuel consumption.^[^ ^45^ ^]^

Furthermore, Air France‐KLM collaborates extensively with various sectors of the aviation industry to accelerate the development of innovative solutions in areas such as aircraft design and maintenance, engines, and synthetic fuels. These solutions are essential for achieving net‐zero emissions goals in aviation. The SBTi is a globally recognized organization that enables businesses to set ambitious emission reduction targets consistent with the latest climate science, in line with the Paris Agreement. SBTi operates in collaboration with organizations like the Carbon Disclosure Project (CDP), the United Nations Global Compact, the World Resources Institute (WRI), and the World Wide Fund for Nature (WWF). SBTi independently evaluates and approves companies' CO_2_ emission reduction targets based on scientific criteria and a scientific approach.^[^ ^47^ ^]^

Air France‐KLM's sustainability goals include reducing emissions per passenger‐kilometer by 30% compared to 2019 levels and transitioning to net‐zero CO_2_ emissions by 2050. To achieve emission reductions, the group is investing in Sustainable Aviation Fuel (SAF). Air France, KLM, and Transavia, which are operated by the group, aim to operate at least five flights to their destinations with a minimum of 30% sustainable aviation fuel. The group's sustainability objectives also encompass social and societal responsibilities. With ≈71 000 employees, the group is committed to combating all forms of discrimination and inequality in the workplace and aims to have women represent 40% of management positions by 2030.^[^ ^45^ ^]^

Air France‐KLM assumed corporate social responsibility obligations and underwent United Nations verification procedures in 2003. The sustainability report of the Air France‐KLM Group was first published in 2014, and in June 2022, they released their 2021 Sustainability Report entirely in digital format. Air France–KLM has established Sustainability Performance Targets and set sub‐goals for both short and long terms. The number of SPTs may vary depending on the Sustainability‐Linked Financing Instrument. Calculations and scope for key performance indicators are determined by the Air France‐KLM Group in accordance with SBTi guidelines.^[^ ^47^ ^]^

#### 4.3.2 Turkish Airlines

Turkish Airlines, founded in 1933 and continuously growing at a rapid pace, placed sustainability at the core of its business model with the announcement of its Sustainability Vision in 2009. During the preparation process of the 2021 Sustainability Report, Turkish Airlines conducted a comprehensive study to identify priority areas. Among the high‐priority areas identified were “Flight Safety and Security, Climate Change, Employee Health and Safety, Customer Expectations and Behavior Change, Fleet Modernization and Development, Digitalization, Business Continuity, Talent and Waste Management”. Additionally, “Business Ethics and Ethical Behavior,” “Compliance with Regulations and Risk Management,” “Customer Satisfaction,” and “Financial Performance” are considered immutable principles of the management approach and were not subjected to prioritization.^[^ ^48^ ^]^

Turkish Airlines has voluntarily participated in performance evaluations by national and international indices and sustainability rating organizations such as DJSI, Financial Times Stock Exchange‐Russell Group Index Series (FTSE4Good), MSCI, EcoVadis, Sustainalytics, Transitions Performance Index (TPI), and the Borsa Istanbul (BIST) Sustainability Index. Turkish Airlines achieved significant scores in these evaluations. In 2021, Turkish Airlines ranked 1st in the “lowest risk” category among 69 participants in the airline sub‐sector, according to the Sustainalytics Environmental, Social and Corporate Governance (ESG) Risk Rating Score, demonstrating its position in the “lowest 1% risk category” in the sector. Turkish Airlines also received a “Bronze” category award in the assessment conducted by Ecovadis. Furthermore, Turkish Airlines earned a place in the BIST Sustainability Index, which includes companies traded on Borsa Istanbul and demonstrates high corporate sustainability performance, in 2021.^[^ ^49^ ^]^

As of the end of 2021, Turkish Airlines reached a fleet of 370 aircraft, with 20 of them being next‐generation wide‐body aircraft, achieving an average fleet age of 8.5 years. In 2021, the airline operated to 333 destinations, serving approximately 1.11 million passengers. The new‐generation aircraft, both narrow‐body and wide‐body, have led to fuel savings of approximately 15% per seat for narrow‐body aircraft and 20–25% for wide‐body aircraft, resulting in a reduction in greenhouse gas emissions. Turkish Airlines continues its efforts to use biofuels, referred to as sustainable aviation fuels. Initially, the airline started using biofuels on flights to various European destinations, with plans to expand the destinations and usage frequency. As part of its R&D activities, Turkish Airlines aims to complete engine tests for Microalgae‐Based Sustainable Bio‐Jet Fuel and then blend it for use in flights.^[^ ^48^ ^]^

In order to mitigate the impact of carbon emissions from flight operations, Turkish Airlines voluntarily committed to implementing the Carbon Offsetting and Reduction Scheme for International Aviation (CORSIA) initiated by the International Civil Aviation Organization (ICAO) as a global solution. As a result, carbon emissions from flights in 2019, 2020, and 2021 were independently verified by a third‐party audit organization. In the coming years, Turkish Airlines aims to offset emissions that exceed baseline emission values using carbon credits obtained from CORSIA‐compliant projects.^[^ ^49^ ^]^

Turkish Airlines has been publishing its sustainability report every year since 2014, and in 2022, it released its eighth report. The preparation of the 2022 Sustainability Report was based on the Global Reporting Initiative (GRI) standards and was prepared in accordance with the Core option. The greenhouse gas emissions reported for the year 2021 in the report were independently verified by a third‐party organization in accordance with the TS EN ISO 14064‐3: 2019 Standard. The selected indicators in the report were determined by PwC Turkey in compliance with the ISAE 3000 (revised) standard.^[^ ^49^ ^]^

A brief summary of indicators Representing the Sustainability Performance of Air France‐KLM and Turkish Airlines is given in **Table** **4**.

**Table 4:** Indicators Representing the Sustainability Performance of Air France‐KLM and Turkish Airlines.

Indicators^a)^		Unit	Air France‐KLM		Turkish Airlines	
			2021	2022	2021	2022
Number of passengers			45000	83000	44800	71800
Number of destinations			310	300	333	342
Fleet average age			12.8	12.1	8.50	8.70
Total number of aircraft			536	522	370	394
Greenhouse gas emissions (Scope 1 GHG protocol)					13463	18170
	Conventional Aviation Fossil Fuel	ktons CO_2_eq	16279	22442		
	Sustainable Aviation Fuel	ktons CO_2_eq	13.3	132.5		
	Ground Operations	ktons CO_2_eq	46.3	45.1		
Greenhouse gas emissions (Scope 2 GHG protocol)					56.4	64.2
	Location Based	ktons CO_2_eq	50.3	50.7		
	Market Based	ktons CO_2_eq	17.8	18.3		
Greenhouse gas emissions (Scope 3 GHG protocol)					3072	4749
	Upstream emissions from fuel production	ktons CO_2_eq	4143	5712		
	Upstream emissions from Sustainable Aviation Fuel	ktons CO_2_eq	3.4	34		
Subtotal carbon emissions (scope 1,2, &3 GHG protocol)		ktons CO_2_eq	20502	28384		
CO_2_eq reduction from SAF (Scope 1 & 3 GHG protocol)		ktons CO_2_eq	9.9	151		
Total Carbon emissions (Scope 1, 2 & 3 GHG protocol)		ktons CO_2_eq	20493	28233	16591	22983
Consumption of raw materials	Conventional Aviation Fuel	ktons	5151	7102	4233	5711
	Sustainable Aviation Fuel	ktons	4.2	42	‐	‐
Fuel efficiency	CO_2_ footprint for passengers transport	grCO_2_eq/passengers/km	95.5	77.0		
	CO_2_ footprint for Cargo transport	gCO_2_eq/100 kg cargo/km	52.9	44.7		
Noise impact	Acoustic performance	%fleet under	70%	75%		
Electricity Consumption	Total consumption	MWh	231903	231542	326487	394003
	Of which renewable	MWh	99357	103456	‐	63921
Water Consumption	Water consumption	m^3^	314200	327666	255238	364508
Non‐carbon emission	NOx	Ktons	0.090	0.188	47	63
Waste Production					1608	2313
Non‐hazardous industrial waste	Total quantity	Tons	9735	14487		
	Percentage recycled	%	44%	44%		
Hazardous industrial waste	Total quantity	Tons	3570	3697		
	Percentage recycled	%	60%	58%		
Total staff (headcount, permanent and fixed‐term contracts)			76803	78950	27532	29520
Ground staff			44810	45325	9938	10510
Cabin crew			23455	24789	12033	13222
Flight Deck Crew			8545	8836	5561	5784
Staff under permanent contract			74664	74049	26519	28404
Recruitment under permanent contract			1825	4140	1007	866
Recruitment under fixed – term contract			2140	6628	6	250
Percentage of women			45.0%	45.8%	46%	47%
Breakdown of staff by age						
29 years			6728	8687	6455	6973
Between 30 and 50 years inclusive			40253	38516	19706	21117
50 Years and above			29822	31747	1371	1529
Absenteeism due to illness			3.20	4.64	1.97	3.34
Frequency rate for workplace accidents			23.46	33.69	7.82	15.58
Total staff with disabilities			1999	1924	212	206

^a)^ This table is prepared by the authors.

The sustainability performance indicators for both airlines for the years 2021 and 2022 are presented in Table 4. The report for Turkish Airlines was created according to Global Reporting Initiative (GRI) standards and presented as a whole on their website.^[^ ^50^ ^]^ In contrast, the report for Air France‐KLM was presented entirely digitally on their website, focusing on specific indicators.^[^ ^45^ ^]^ The representative indicators included in Table 4 were considered to allow for a comparison of the sustainability performance of both airlines. The values for Air France‐KLM were taken from the Universal Registration Document 2022^[^ ^47^ ^],^ which offers a more comprehensive approach rather than a sustainability report.

The most significant difference identified between the sustainability reporting of the two airlines is that Turkish Airlines provides data for the past 5 years just to include current status, while Air France‐KLM presents both the current status and the targeted status for certain representative indicators for various years (2030, 2040, and 2050). This difference arises from the use of different standards or frameworks for presenting sustainability performance, resulting in significant variations in the content, volume, and depth of detail in the reports.

It is important to note that there are no mandatory or legally binding standards or frameworks for the preparation of sustainability reports for airline companies, and the chosen reporting format can vary between companies. At the international level, the reporting framework established by the GRI has emerged as almost a standard. GRI, an independent non‐profit organization, published the world's first recognized framework for sustainability reporting in 2000, and a continuously updated and expanded set of guidelines eventually became the “GRI Standards” in 2016, widely regarded as the most comprehensive sustainability reporting standards in the world. Among the examples, Turkish Airlines reports in compliance with GRI standards^[^ ^45^ ^]^ whereas Air France‐KLM publishes sustainability reports in an independent format. The independently published reports of Air France‐KLM are quite comprehensive and meet GRI standards with the exception of GRI content index.

When the performance values are focused in Table 4, especially regarding total carbon emissions, in Air France‐KLM displays a 30% reduction in 2022 compared to 2019, while Turkish Airlines shows a 22% increase during the same period. This difference stems from the fact that Air France‐KLM initiated its sustainability efforts earlier and took significant steps. For example, KLM started its sustainability reporting in the 1996/1997 fiscal year, which initially began as an environmental report and later evolved into sustainability reports covering a broader range of sustainability topics. KLM is one of the exemplary airlines that focus on sustainability in various operational areas. Similarly, Air France has been publishing environmental reports since 1996 and has been gradually expanding its sustainability approach since partnering with KLM in 2004.

The increased carbon emissions of Turkish Airlines compared to 2019 are attributed to the company's growth. However, it is expected that emissions will show a decreasing trend in the near future due to initiatives such as fleet modernization and alternative fuel research. Both airlines have clear goals in their sustainability reports to enhance environmental performance. Emphasizing these goals reflects the close relationship between ecological and economic efficiency. For example, reducing fuel consumption or transitioning to alternative fuels can contribute to emission reduction while also reducing operating costs.

In addition to all these indicators, another indicator that has not been considered yet but is thought to be necessary is ‘training facilities adopting Extended Reality (XR) Augmented Reality (AR) and Virtual Reality (VR) technologies’. Considering the expensive nature of the aviation sector and the high cost of errors, the importance of VR and AR technologies is increasingly being recognized by aviation companies for providing better services and more accurate personnel training. While there have been reductions in casualties resulting from aviation accidents and incidents, significant steps are still required. To this end, the use of these technologies in the training of airline crews is expected to make the industry safer and more cost‐effective. The primary use cases for these technologies include “aircraft inspection training, cabin crew training, flight deck training, aircraft maintenance, and aircraft, maintenance, and repair”. Among the companies that utilize these technologies, Çelebi Aviation Holding in Turkey has established a VR‐based Aviation Academy, and Air France is another notable example. Additionally, airlines such as Emirates, Qatar Airways, Lufthansa, Japan Airlines, and others also make use of these technologies. Given that these technologies are expected to have a lasting impact on the aviation industry, it is recommended that they be included as parameters for evaluating sustainability performance in the aviation sector.

## 5 Policy Proposals for Promoting Sustainable Aviation in Turkey

In this study, the aim is to determine the stage of sustainability studies in the Turkish civil aviation sector, which has experienced rapid growth and development in recent years. The study also seeks to assess the extent to which these studies align with global sustainable aviation policies. Based on the results, policy recommendations have been formulated to address the identified deficiencies in the current situation. The feasibility of these proposed solutions in the short, medium, and long term has also been evaluated. In this study, the short term refers to a time period of up to 5 years, the medium term covers 5 to 10 years, and the long term spans 10 years or more. The analysis of the current situation in Turkey indicates that the rapidly growing aviation sector is particularly successful in keeping up with the latest technologies such as the new aircraft models (ex. A350‐900, Airbus A321neo, B787‐9 Dreamliner,) included in the aircraft fleets of airline companies in Turkey labeled as low‐emission, fuel‐efficient and with noise control; and the usage of new aircraft fuels of Sustainable aviation fuels.^[^ ^48^, ^51^ ^]^ With these new technologies, we can observe fuel savings of 15% per seat in the new generation narrow‐body aircraft, fuel savings of 20–25% in wide‐body aircraft, and a parallel reduction in flight‐related greenhouse gas emissions (with 37 082 tons of aviation fuel saved and 116 808 tons of carbon emission is prevented) in 2021 with fleet modernization in 2021.^[^ ^48^ ^]^

This success is primarily driven by the private sector's efforts to enhance international competitiveness. The young age of the aircraft fleet contributes significantly to reducing emission consumption. However, as the sector continues to grow, emission consumption also increases. Turkey demonstrates enthusiasm and success in aligning with international aviation trends to sustain its achievements in the industry.

Deficiencies have been identified in several categories, including economic, political, social, and infrastructural aspects. From an economic and technical standpoint, the rapid growth of civil aviation in Turkey naturally leads to an increase in carbon emissions. Achieving environmental sustainability would require halting aviation growth, which may not be economically feasible. As an alternative, proposals from the IPCC 2022 report are considered, such as transitioning to low‐carbon biofuels or synthetic fuels, using electric energy for short‐haul flights, and increasing the use of high‐speed rail for distances between 400–800 km. Encouraging the use of railways for relatively short distances is recommended in Turkey, but this will require balancing ticket prices for both rail and air travel. Expanding railway networks for both passenger and freight transport and establishing closer cooperation with the aviation sector may take longer to implement.

Transitioning to the use of low‐carbon biofuels is being researched in Turkey, but it is expected to be feasible in the medium term. However, faster progress is needed to keep up with successful examples like Air France‐KLM, which conducted its first biofuel flight to Paris in 2011 and to New York in 2013. The adoption of electric energy for short‐haul flights in Turkey is likely to take place in the medium term.

In conclusion, addressing sustainability challenges in civil aviation will require a comprehensive approach that includes a mix of short‐, medium‐, and long‐term solutions. Balancing economic, environmental, and social considerations will be crucial in achieving sustainable aviation in Turkey.

The cooperation of various actors in sustainable aviation is of great importance. The responsibilities of all organizations regarding the environment should be clearly defined, and continuous coordination and cooperation between public, sectoral, sub‐sectoral, and non‐governmental actors should be ensured. While sustainability studies in aviation in Turkey are advancing primarily through the private sector, the government plays a crucial role in creating a framework to eliminate uncertainty through policies, including legislation and/or harmonization, and providing direct financial support such as tax reductions and research grants.^[^ ^52^ ^]^ A study on sustainability in civil aviation in the United Kingdom by McManners^[^ ^52^ ^]^ emphasized that one major obstacle to long‐term sustainable strategies is the impact of political cycles, such as elections, on the process. Thus, it is recommended that such strategies be established as high‐level policies and be insulated from the political process.^[^ ^52^ ^]^ It is crucial that such strategies are embraced as high‐level policies in the short term in Turkey.

Considering inter‐sectoral cooperation is essential when formulating policies. Tourism, which constitutes a significant portion of air transportation passengers, is an important input for the sustainability of the aviation industry. Similarly, the continuity of tourism largely depends on the aviation sector. Therefore, environmental policies intended for the aviation sector should also be designed in a way that aligns with the tourism sector.

In Turkey, it has been determined that there is insufficient regulation on sustainability in national legislation. Inadequate regulations and discrepancies between national and international legislation pose obstacles to accelerating progress in this area. However, a positive development occurred while working on this paper, as Turkey signed the Paris Agreement. The harmonization of the Convention's regulations with national legislation is expected to accelerate the sustainability process. Therefore, completing the missing legislation in Turkey is necessary in the short term. At this point, policy transfers from international organizations' best practices and policies gain importance for Turkey. Making carbon emission trading compulsory in the short term, even though it is not yet mandatory in Turkey, can lead to a more effective carbon balancing policy through inter‐sectoral cooperation.

Apart from these, consciousness and awareness studies are considered to be of great importance. Sustainability awareness should primarily be fostered within society, becoming a culture that influences the consumption habits of the people. However, our findings reveal that in Turkey, this consciousness primarily started within the sector due to international obligations, and efforts have been made to disseminate it to society through sectoral initiatives. In Turkey, it is essential to give more prominence to sectoral environmental studies and scientific research, both within civil society and universities. The scarcity of scientific research in this field, as highlighted in this study, can also be interpreted as a lack of environmental awareness. People's adoption of environmental values has been shown to impact their travel habits. Studies indicate that individuals who care about the environment tend to drive less or choose environmentally friendly cars.^[^ ^53^ ^]^ Additionally, consumers are more likely to purchase energy‐efficient devices when energy efficiency labels are displayed on products.^[^ ^54^ ^]^ Information policies can also contribute to changing social norms related to travel and consumption decisions. Given this situation, it is recommended to mandate the declaration of flight emissions in the short term.

In Turkey, infrastructure‐related problems manifest as both financial issues and a shortage of qualified human resources. The allocated financing for sustainability varies, influenced by the country's other developmental priorities and the changing policy preferences of the government. To effectively maintain the international presence of Turkey's rapidly growing aviation sector, it is crucial to allocate sufficient financial resources to this field in the short term. Qualified human resources can be developed in the short term through training support provided by international aviation organizations and in the medium term through regulations in the civil aviation departments of universities.

Turkish Airlines and AirFrance KLM Comparison highlighted out two main conclusions for global sustainability challenges: 1) The lack of standardization for effective comparison of sustainability aviation practices and 2) The lack of data related to any decarbonization indicators in aviation (such as carbon emissions, water consumption, waste management, fuel efficiency, fleet modernization, etc.) and concrete decarbonization targets for future projections for sustainable aviation practices. Since there is an urgent need for road maps and strategy document to realize these specific policy recommendations, these policy proposals should have implemented in short‐run.

As summarized above, it has been concluded that some of the deficiencies identified for Turkey are related to environmental awareness, resource problems, technical challenges, and economic reasons. If prioritization is to be made, the essence of the deficiency lies in consciousness and awareness. Developing a culture of environmental awareness within society requires coordinated efforts and contributions from all actors, including civil society, the public, and the aviation sector. When necessary awareness is instilled in society, institutional behaviors to protect the environment become inevitable, as they will also reflect on consumption and decision‐making habits. In this regard, the importance of creating a network chain and fostering cooperation among actors is once again emphasized in the study. The recommendations described above are summarized in **Table** **5**.

**Table 5:** Policy proposals for sustainable aviation in Turkey.

Theme	Problem	Policy Recommendations	Term
Economic	Difficulties in implementation of EU ETS Systems	Giving financial incentives to airline companies and other aviation organizations that carry out sustainable aviation studies	Short
		Switching to low carbon biofuels or synthetic fuels	Medium
		Expanding the railway networks	Long
Political	Compatibility to international Agreements (such as Paris Agreement, Green Deal)	Adopting sustainability strategies should as high‐level policies	Short
		Gather emission management policies under a single organization	Short
		Transferring and harmonizing the relevant legislations	Short
		Following and transferring current and functional policies and practices in the international sector	Short, Medium
		Creating action plans and updating, at regular intervals	Short, Medium, Long
		Making carbon emissions trading mandatory	Short
		Following more effective carbon balancing policy through intersectoral cooperation	Short
		Following balanced growth policies among transportation sectors by cooperating with other transportation sectors (such as railway transportation)	Short
Social	Lack of awareness about sustainable aviation practices	Raising awareness of the whole society with educational tools	Short, Medium, Long
		Raising scientific research in the field of civil society and in universities	Short
		Changing social norms in travel decisions by mandating the declaration of flight emissions	Short
		Raising sectoral sustainability studies	Short
Infrastructural/Institutional	Cooperation between the public and private sector	Switching to low‐carbon biofuels or synthetic fuels	Medium
		Expanding the railway networks	Long
	Lack of human resources	Increasing scientific studies on the subject, especially in universities	Short
	Lack of Standardization for effective comparison of sustainability aviation practices	Compliance with international standards for the selection and evaluation of sustainability indicators and preparation of sustainability reports	Short
	Lack of data related to the emissions and concrete targets for future projections	Holistic approach to data collection, generation, and analysis for sustainability indicators reporting, and putting targets	Short

## 6 Conclusion 

In this study, the main aim was to determine the current status of sustainability studies in the Turkish civil aviation sector and to what extent it aligns with global sustainable aviation policies. Additionally, policy recommendations were developed to address the identified deficiencies in field research. The obtained results can be summarized as follows:

- The analysis of the current situation in Turkish sustainable civil aviation shows that Turkey is particularly successful in adopting the latest technologies and is eager to catch up with international trends. However, further steps are still needed.
- According to the interview results, the shortcomings are mainly concentrated in areas such as compatibility with international agreements, national legislation, incentives, obligations, and environmental awareness.
- The most urgent suggestion to be made by the government is to consolidate emission management policies under a single organization and harmonize relevant legislation.
- The most urgent suggestion to be made by the private sector is the implementation of international obligations, such as carbon trading.
- The most urgent suggestion is to put concrete and specific targets for future projections in short term by following a holistic and multidimensional approach to data collection and data analysis for international sustainability indicators reporting to make effective comparison and improvements.

The present study represents a scientific endeavor aimed at fostering the advancement of the global civil aviation sector, with a particular focus on its development within Turkey. It is anticipated that this research will provide valuable insights for future scholars. In light of potential forthcoming investigations, it is suggested to assess the effectiveness of sustainability initiatives, currently popular in management practices, within the sustainable aviation realm. This assessment should distinguish between genuine effectiveness and strategies primarily serving as marketing tactics to gain legitimacy. Furthermore, potential research areas include an examination of whether airlines recommended in this study can implement cost‐saving measures in their flight operations, the reasons behind such decisions, and the formulation of necessary roadmaps if they choose this as a strategic direction. Another prospective research topic involves evaluating the impact of sustainable aviation practices on various sectors, aiming for balanced economic growth, inter‐sectoral collaboration, and division of labor. Additionally, within the framework of the Sustainable Development Goals, it is suggested to separately evaluate the recommendations provided in this study and preliminarily assess their contributions to specific development objectives. Exploring these recommendations within the context of the Sustainable Development Goals presents an avenue for future research development in this field.

## Conflict of Interest

The authors declare no conflict of interest.

## Author Contributions

All authors contributed to the study conception and design. Data collection and analysis were performed by U.T., Y.E.‐T., and B.H.G.‐H. The first draft of the manuscript was written by B.H.G.‐H. and the other authors commented on previous versions of the manuscript. This paper is based on N. U. Temel's MSc. Thesis titled “Evaluation of Turkey's civil aviation policy within the scope of the global sustainable aviation” from Marmara University (TR) Graduate School of Social Sciences.

## Ethics Approval

The field research for Empirical Analysis was conducted under the Ethical Approval of Marmara University Social Sciences Research Ethical Council no: 2021‐43 (Dated to October 25, 2021).

## Data Availability

The data that support the findings of this study are available on request from the corresponding author. The data are not publicly available due to privacy or ethical restrictions.
